# Supplementary material for: Reward-related self-agency is disturbed in depression and anxiety
Source: PLoS One. 2023 Mar 15;18(3):e0282727. doi: 10.1371/journal.pone.0282727 (PMC10016695; doi:10.1371/journal.pone.0282727)
Supplement: S1 Table — (DOCX) [file pone.0282727.s001.docx]

**Supporting Information**

**S1 Table.**

| Independent | Predictor | Estimate | SE | t-value | P |
| --- | --- | --- | --- | --- | --- |
| Rating | **Intercept** | 2.65 | 0.13 | 20.76 | <0.001 |
|  | Group - DA | 0.01 | 0.18 | 0.04 | 0.972 |
|  | **Agency (*Ambiguous*)** | 0.40 | 0.09 | 4.35 | 0.000 |
|  | **Agency (*Computer*)** | 1.16 | 0.15 | 7.82 | 0.000 |
|  | **Feedback (*Win*)** | -0.66 | 0.11 | -6.04 | 0.000 |
|  | Group (*DA*) x Agency (*Ambiguous*) | 0.22 | 0.16 | 1.36 | 0.175 |
|  | Group (*DA*) x Agency (*Computer*) | -0.05 | 0.23 | -0.20 | 0.842 |
|  | Group (*DA*) x Feedback (*Win*) | 0.27 | 0.11 | 2.46 | 0.014 |

Results of the Linear Mixed Effects Model to test agency and feedback as predictors of rating (sense of agency) for patients with Depression and Anxiety Disorder (DA).
