# Supplementary material for: Reward-related self-agency is disturbed in depression and anxiety
Source: PLoS One. 2023 Mar 15;18(3):e0282727. doi: 10.1371/journal.pone.0282727 (PMC10016695; doi:10.1371/journal.pone.0282727)
Supplement: S2 Table — (DOCX) [file pone.0282727.s002.docx]

**Supporting Information**

**S2 Table.**

| **Independent** | **Predictor** | **Estimate** | **SE** | **t-value** | **P** |
| --- | --- | --- | --- | --- | --- |
| Rating | **Intercept** | 2.65 | 0.13 | 20.72 | <0.001 |
|  | Group (*GAD*) | 0.01 | 0.21 | 0.05 | 0.96 |
|  | Group (*MDD)* | 0 | 0.25 | 0.02 | 0.985 |
|  | **Agency (*Ambiguous*)** | 0.4 | 0.09 | 4.4 | <0.001 |
|  | **Agency (*Computer*)** | 1.16 | 0.15 | 7.81 | <0.001 |
|  | **Feedback (*Win*)** | -0.66 | 0.11 | -6.09 | <0.001 |
|  | Group (*GAD*) x Agency (*Ambiguous*) | 0.13 | 0.19 | 0.72 | 0.473 |
|  | Group (*MDD*) x Agency (*Ambiguous*) | 0.38 | 0.23 | 1.66 | 0.097 |
|  | Group (*GAD*) x Agency (*Computer*) | -0.09 | 0.26 | -0.33 | 0.738 |
|  | Group (*MDD*) x Agency (*Computer*) | 0.01 | 0.33 | 0.04 | 0.969 |
|  | Group (*GAD*) x Feedback (*Win*) | 0.23 | 0.12 | 1.91 | 0.056 |
|  | **Group (*MDD*) x Feedback (*Win*)** | 0.38 | 0.16 | 2.39 | 0.017 |

Results of the Linear Mixed Effects Model to test agency and feedback as predictors of rating (sense of agency) for patients with primary Generalized Anxiety Disorder (GAD) and Major Depressive Disorder (MDD), which compromised the Depression and Anxiety Disorder (DA) group.
