# Supplementary material for: Reward-related self-agency is disturbed in depression and anxiety
Source: PLoS One. 2023 Mar 15;18(3):e0282727. doi: 10.1371/journal.pone.0282727 (PMC10016695; doi:10.1371/journal.pone.0282727)
Supplement: S3 Table — (DOCX) [file pone.0282727.s003.docx]

**Supporting Information**

**S3 Table.**

| **Independent** | **Predictor** | **Estimate** | **SE** | **t-value** | **P** |
| --- | --- | --- | --- | --- | --- |
| Rating | **Intercept** | 2.0074 | 0.040489 | 49.579 | <0.001 |
|  | **Feedback** | 1.1045 | 0.064793 | 17.047 | 1.78E-64 |
|  | **Agency** | 0.39541 | 0.025456 | 15.533 | 6.13E-54 |

Results of the Linear Mixed Effects Model to test agency (self, ambiguous and computer) and feedback (positive-win and negative-loss) as predictors of rating (sense of agency) for the online sample.
